# Blast-induced cochlear synaptopathy in chinchillas

**DOI:** 10.1038/s41598-018-28924-7

**Published:** 2018-07-16

**Authors:** T. T. Hickman, C. Smalt, J. Bobrow, T. Quatieri, M. C. Liberman

**Affiliations:** 1 Eaton-Peabody Laboratories, Massachusetts Eye and Ear, Boston, MA 02114 USA; 2 Department of Otolaryngology, Harvard Medical School, Boston, MA 02115 USA; 3 Bioengineering Systems and Technologies, MIT Lincoln Laboratory, Lexington, MA 02421 USA

## Abstract

When exposed to continuous high-level noise, cochlear neurons are more susceptible to damage than hair cells (HCs): exposures causing temporary threshold shifts (TTS) without permanent HC damage can destroy ribbon synapses, permanently silencing the cochlear neurons they formerly activated. While this “hidden hearing loss” has little effect on thresholds in quiet, the neural degeneration degrades hearing in noise and may be an important elicitor of tinnitus. Similar sensory pathologies are seen after blast injury, even if permanent threshold shift (PTS) is minimal. We hypothesized that, as for continuous-noise, blasts causing only TTS can also produce cochlear synaptopathy with minimal HC loss. To test this, we customized a shock tube design to generate explosive-like impulses, exposed anesthetized chinchillas to blasts with peak pressures from 160–175 dB SPL, and examined the resultant cochlear dysfunction and histopathology. We found exposures that cause large >40 dB TTS with minimal PTS or HC loss often cause synapse loss of 20–45%. While synaptopathic continuous-noise exposures can affect large areas of the cochlea, blast-induced synaptopathy was more focal, with localized damage foci in midcochlear and basal regions. These results clarify the pathology underlying blast-induced sensory dysfunction, and suggest possible links between blast injury, hidden hearing loss, and tinnitus.

## Introduction

Hearing impairment due to blast- or impulse-noise exposure is an increasingly common casualty among military personnel and civilians^1–6^. In addition to the well-known effects of acoustic injury on auditory sensitivity, as assessed by the threshold audiogram, perceptual anomalies such as tinnitus or hyperacusis, and difficulty understanding speech in a noisy environment, are often associated with blast-induced damage^1,7–9^. These perceptual anomalies can be present, and highly bothersome, even in cases where threshold sensitivity has returned to normal^7^.

Recent work on continuous-noise exposures in animal models has revealed that many of the synaptic connections between inner hair cells (IHCs) and auditory-nerve fibers can be permanently destroyed in cases where the noise-induced threshold shifts, and hair cell damage, are completely reversible^10^. This type of cochlear synaptopathy has now been demonstrated in mice^10^, guinea pigs^11^, chinchillas^12^, monkeys^13^, and humans^14^. This primary neural degeneration has been termed “hidden hearing loss,” because it does not affect threshold sensitivity until it exceeds ~80%^15^, and therefore can hide behind the audiogram, whether thresholds are normal or elevated. It may, however, lead to problems understanding speech in noisy environments due to loss of information channels, and may also be a key elicitor of tinnitus and/or hyperacusis, due to subsequent compensatory changes in “central gain”^16^. In animal models of cochlear synaptopathy, noise-exposure intensities are often adjusted to be at the border between temporary and permanent threshold shifts. Although all exposures that destroy hair cells, and thus cause permanent threshold shifts (PTS), may also cause synaptopathy on surviving hair cells, not all exposures causing only temporary threshold shifts (TTS) produce synaptopathy^17^.

Prior work has shown that impulse-noise exposures can cause devastating damage to the cochlear sensory epithelium, including massive and widespread hair cell death^18–20^. More recent studies have documented the presence of cochlear synaptopathy in IHCs remaining after blast exposure, but only after exposures that also caused significant PTS and hair cell damage^21,22^.

The aim of the present study was to determine if cochlear synaptopathy also occurs after blast exposures that produce only TTS and therefore also minimal hair cell damage, i.e. to determine if, for blast exposures, as for continuous-noise exposures, the synaptic connections between hair cells and cochlear nerve fibers are more vulnerable than the hair cells themselves. The answer is relevant to hypotheses about the mechanisms underlying noise-induced synaptopathy, as well as to speculations about the genesis of tinnitus and hyperacusis after blast-induced trauma. We chose to study the chinchilla because most of the pioneering work on the effects of impulse noise on the auditory periphery were performed on a chinchilla model, due in part to its human-like hearing range, low-impedance tympanic membrane, and large ear canal^19,20,23–25^.

## Methods

### Animals and Groups

Female chinchillas, aged 6–9 months, were assigned to one of the following groups: unexposed controls (n = 9 animals), one blast @ 160 dB SPL (n = 2, mean = 161.6 ± 1.27 (standard deviation) dB SPL or 81.3 dB-LA_eq8hr_), one blast @ 175 dB (n = 11, mean = 174.9 ± 1.94 dB SPL or 91.7 dB-LA_eq8hr_), 10 blasts @ 165 dB (n = 8, mean = 165.8 ± 1.94 dB SPL or 94.0 dB-LA_eq8hr_), 5 blasts @ 175 dB (n = 1 animal, mean = 174.6 dB ± 0.56 dB SPL or 99.0 dB-LA_eq8hr_), or 10 blasts @ 175 dB (n = 1, mean = 174.7 ± 0.45 dB SPL or 102.6 dB-LA_eq8hr_). Blast levels are reported as peak pressure, while the energy of the blast(s) is indicated by the 8-hour A-weighted Energy (dB-LA_eq8hr_). The mean A-duration, i.e. the time between the blast pressure onset and first zero-crossing, for all blasts was 1.44 ms (SD = 0.64 ms). Interblast interval was 1–5 minutes. Cochlear function was either assessed immediately after blast exposure (n = 2 animals), or at 1 wk post exposure (all other animals), and cochleas were removed for histological analyses. Animals were anesthetized, and core temperature was maintained at 37–38 °C during all exposures and testing. All procedures were approved by the IACUC of the Massachusetts Eye and Ear, and the investigators adhered to the “Guide for Care and Use of Laboratory Animals” as prepared by the Committee on Care and Use of Laboratory Animals of the Institute of Laboratory Animal Resources, National Research Council.

### Blast Exposures

**Figure 1:**
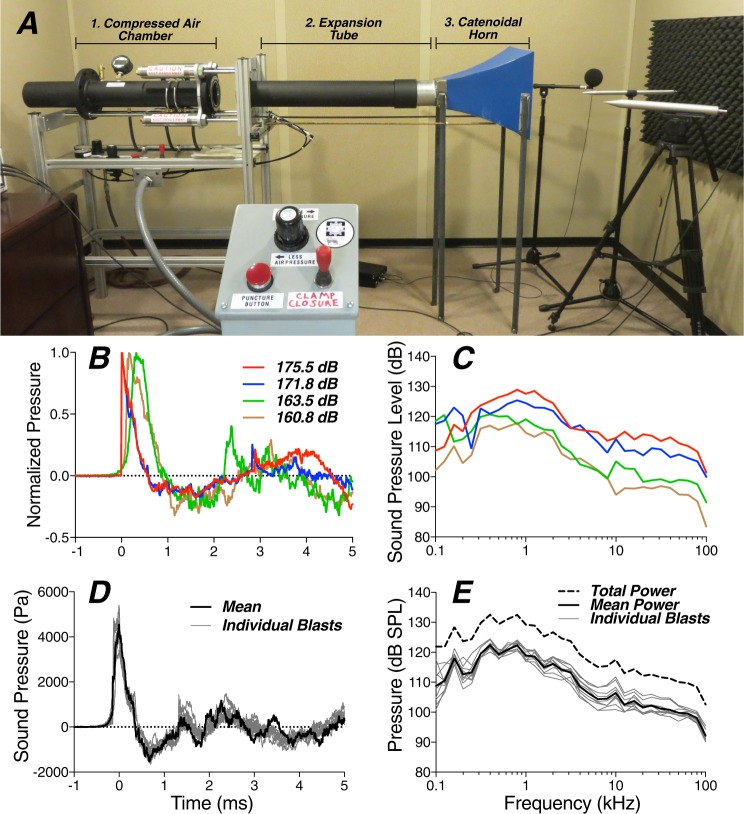
Structure and performance of the custom shock tube. (**A**) Our shock tube consists of a compressed air chamber (1), separated from an expansion tube (2), and catenoidal horn (3) by a thin Mylar or brass membrane (not shown) with a remotely actuated puncture pin. In the image, the air chamber has been pulled away from the expansion tube, and no membrane is in place. The total length of the apparatus, including the horn is 6′6″. When the air chamber is filled to 27 psi, peak pressures near 175 dB SPL can be reliably produced at the orifice of the horn. (**B**) Example pressure waveforms measured above the animal’s head (at the midline) for levels spanning the range tested and arbitrarily aligned by placing time 0 at 5% peak pressure. (**C**) Example spectra obtained from pressure waveforms, as measured at the tragus, from blasts spanning the range of peak pressures. (**D,E**) Pressure waveforms (peak-aligned) and resultant spectra measured near the tragus during 10 consecutive blasts @ 165 dB SPL peak.

Our shock tube, based on a NIOSH design^26^, is small enough for use in an audiometric sound booth, yet powerful enough to generate broad-spectrum (0.3–100 kHz) acoustic pressure waves up to 175 dB SPL peak pressure (Fig. 1). The shock tube was constructed by Mark Cauble (B/C Precision Tool Inc.). By varying compression pressures, membrane materials, and membrane thickness, blasts from 160–175 dB SPL peak pressure can be reliably generated. Static pressure in the shock-tube pressure chamber was measured using an Omega PX309. The blast pressure was measured with two GRAS 46DP 1/8″ microphones placed near the tragus, one on each ear. A PCB 137A23 blast-pressure pencil probe was positioned above the animal, closer to the horn of the shock tube to capture the initial peak pressure without any secondary reflections from the animal or the platform. This recording configuration was intended to capture the free-field incident pressure wave, the characteristics of which are typically used to quantify exposure, as well as a measurement as close to the ear as possible, which may be more representative of the true dose^27^. All signals were acquired and digitized with a National Instruments PXI-4462 at a 200 kHz sampling rate. Animals were anesthetized with ketamine/xylazine during exposure, which was carried out in a soundproofed chamber maintained near 33 °C.

### Cochlear Function Tests

Chinchillas were anesthetized with xylazine and ketamine, the bullas opened, and the external ear canal removed. Compound action potentials (CAPs) were recorded with a silver wire on the cochlear capsule near the round window, and an intramuscular ground electrode at the vertex. CAPs at each sound pressure level (1 dB steps) and frequency (half-octave spacing from 1–16 kHz) were averaged from 32 5-msec tone pips (0.5 ms rise-fall) presented at 20/sec in alternating polarity. Threshold was defined as the sound pressure required to produce a 5 μV peak-to-peak response. Distortion product otoacoustic emissions (DPOAEs) at 2f1-f2 were measured with primary tones in a ratio f2/f1 = 1.2 and at levels L1 = L2 + 10 dB. DPOAE threshold was computed by interpolation as the f1 level required to produce a DPOAE of 5 dB SPL.

### Cochlear Processing

Cochleas were fixed by transcardial perfusion with 4% paraformaldehyde, while the animal was anesthetized with ketamine and xylazine. After perfusion, cochleas were extracted, the scalae were flushed with fixative through the round and oval windows, and the cochleas were post-fixed for 2 hrs. Ears were then decalcified in EDTA, dissected into half-turns and permeabilized. Tissue was permeabilized by freezing on dry ice, incubated for 1 hr at room temperature in blocking solution (PBS with 0.3% Triton X, 5% normal horse serum, and 0.02% sodium azide), incubated overnight at 37 °C, as previously described^28–30^, in primary antibodies: (1) mouse isotype IgG1 anti-C-terminal binding protein 2 (CtBP2, 1:100, BD Transduction Laboratories #612044), (2) rabbit anti-espin (ESPN, 1:100, Sigma #HPA028674) and (3) mouse isotype IgG2 anti-glutamate receptor 2 (GluA2, 1:2000, Millipore #MAB397). Primaries were followed by 2-hr incubations at 37 °C with secondary antibodies: (1) goat anti-mouse IgG1 Alexa Fluor 568 conjugate (1:1000, Thermo Fisher #A-21124), (2) goat anti-mouse IgG2a Alexa Fluor 488 conjugate (1:500, Thermo Fisher #A-21131) and (3) biotinylated donkey anti-rabbit FAB fragment (1:400, Jackson ImmunoResearch #711-067-003), and a 1-hr incubation in streptavidin-conjugated Alexa Fluor 647 (1:200, Thermo Fisher #S-32357) tertiary label. Following tertiary staining, rabbit anti-myosin VIIa (1:100, Proteus BioSciences #25–6790) was incubated overnight at 37 °C, followed by a 2-hr incubation at 37 °C in goat anti-rabbit Pacific-Blue-conjugated antibody (1:200, Thermo Fisher #P-10994). All antibodies were diluted in PBS containing 1% normal horse serum, 0.3% Triton X, and 0.02% sodium azide. We added no anti-rabbit blockers between the initial rabbit anti-ESPN step and the final rabbit anti-myosin step, because we have no need to differentiate anti-myosin hair-cell-cytoplasm labeling from anti-myosin stereocilia labeling.

### Innervation Analysis and Hair Cell Counts

Cochlear lengths and a frequency-place map were obtained for each case from low-power widefield images of the myosin channel in each dissected piece using an imageJ plugin (https://www.masseyeandear.org/research/otolaryngology/investigators/laboratories/eaton-peabody-laboratories/epl-histology-resources). To aid in hair cell counting, the plug-in superimposes lines dividing the cochlear spiral into 50 equal segments from apex to base, and the fractional survival computed within each segment. To count synapses, confocal z-stacks (two adjacent fields at each cochlear region) were collected using a 63x glycerol-immersion objective (N.A. = 1.3) and 2.41X zoom on a Leica TCS SP8 confocal at cochlear frequency locations corresponding to the physiological assays. Similar excitation and detection parameters were used in all imaged samples. Synaptic ribbons (CtBP2-positive puncta) in the IHC area were counted using Amira (Visage Imaging), and the xyz coordinates were fed to custom re-projection software to assess the fraction of ribbons with closely apposed glutamate-receptor patches (i.e. GluA2 puncta)^28^.

### Data availability

The datasets generated and analyzed in this study are available from the corresponding author on request.

## Results

### Overview

We exposed anesthetized chinchillas bilaterally to blast waves using a shock tube pressurized to 12–27 psi, then released by puncturing a thin Mylar® or brass membrane with a remotely actuated puncture pin. As shown in Fig. 1, when directed through an expansion tube and a catenoidal horn, these blast waves produced rapid pressure transients (Fig. 1B,D) with relatively flat spectra (Fig. 1C,E). During exposure, the animal was held with a mouth bar and positioned facing the horn, with condenser microphones close to the tragus on each side. Peak pressures were similar between the two ears: interaural differences averaged 1.2 dB and were always <3.2 dB. When multiple exposures were presented, the interblast time was initially set to 5 minutes, since replacing the membrane and inspecting the animal was required between each blast. In later experiments, the interblast interval was reduced to 1 minute. Results at the two interblast intervals were not significantly different: p values ≥ 0.19 by repeated-measures 2-way ANOVAs for both physiological measures (CAP and DPOAE thresholds) and histological measures (hair cell loss and synaptic loss).

In pilot experiments, we attempted to measure cochlear function before and immediately after exposure using minimally invasive techniques (i.e. auditory brainstem responses and DPOAEs). However, pre-exposure cochlear thresholds were often unstable, likely due to middle-ear pressure changes arising from anesthesia-induced loss of normal eustachian-tube function and an unvented middle-ear space^31^. Thus, we changed the experimental design to one in which cochlear function was tested only in an open-bulla terminal experiment, just before tissue harvest, and in which we exposed the animals as soon as possible after anesthetization, to avoid build-up of pressure difference across the tympanic membrane.

**Figure 2:**
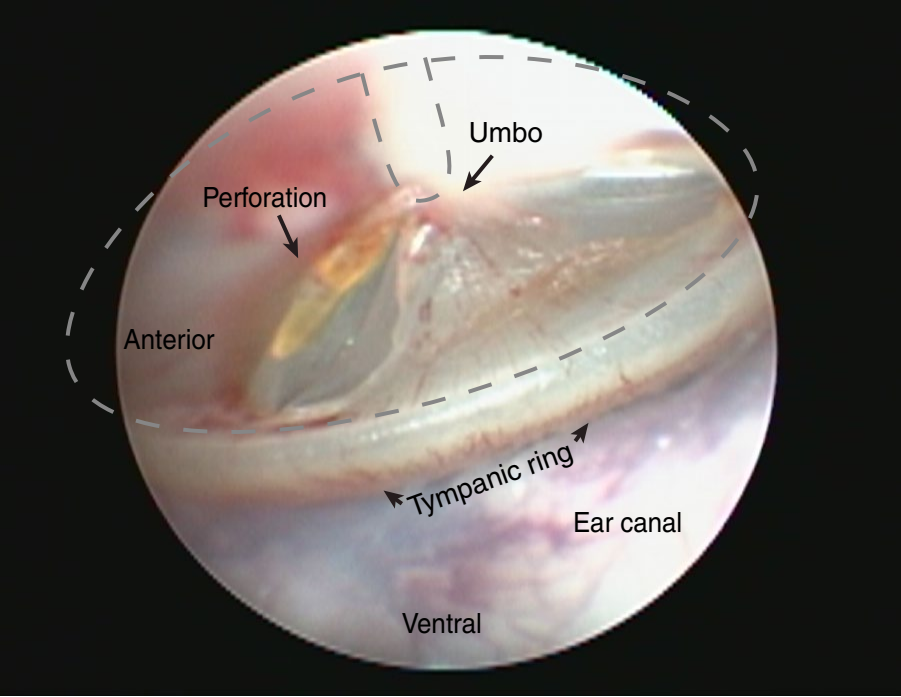
A large eardrum rupture seen after a single 175 dB blast. After each blast, we examined the ear canal and eardrum with an otoscope. Eardrum rupture was never seen after 160 dB or 165 dB blasts, but was sometimes seen after a single 175 dB blast (4/20 ears), and always seen after 5–10 consecutive 175 dB blasts (4/4). Ruptures were usually small tears in the inferior pars tensa, and a small amount of bleeding was often visible.

Immediately after exposure, we examined the eardrum bilaterally with an otoscope. Tympanic-membrane rupture was sometimes seen after single blasts, but only at the highest peak SPL (175 dB: 4/20); one example is shown in Fig. 2. Membrane rupture was always seen after multiple (5 or 10) blasts @ 175 dB (4/4), but was never seen after single or multiple exposures at lower SPLs (160 or 165 dB: 0/4 and 0/16 respectively).

### Histopathology

**Figure 3:**
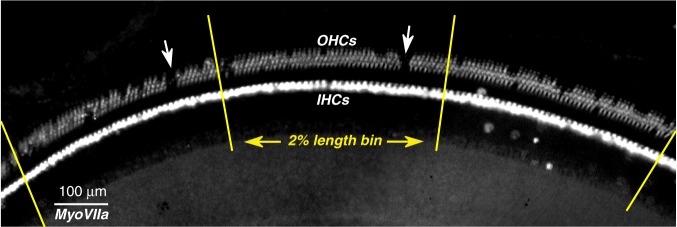
Low-power image of the type used to assess hair cell survival. Widefield image centered on the 13 kHz region of an ear exposed to 10 blasts @ 165 dB SPL peak. Yellow lines illustrate the boundaries used to bin the hair cell counts, corresponding to 2% increments of cochlear length. White arrows point to two regions where all three rows of OHCs are missing. OHC loss was 9.3% when averaged between the central 2% segment shown here and the segment immediately to its right: bin widths in Figs 4 and 5 are 4% of cochlear length.

**Figure 4:**
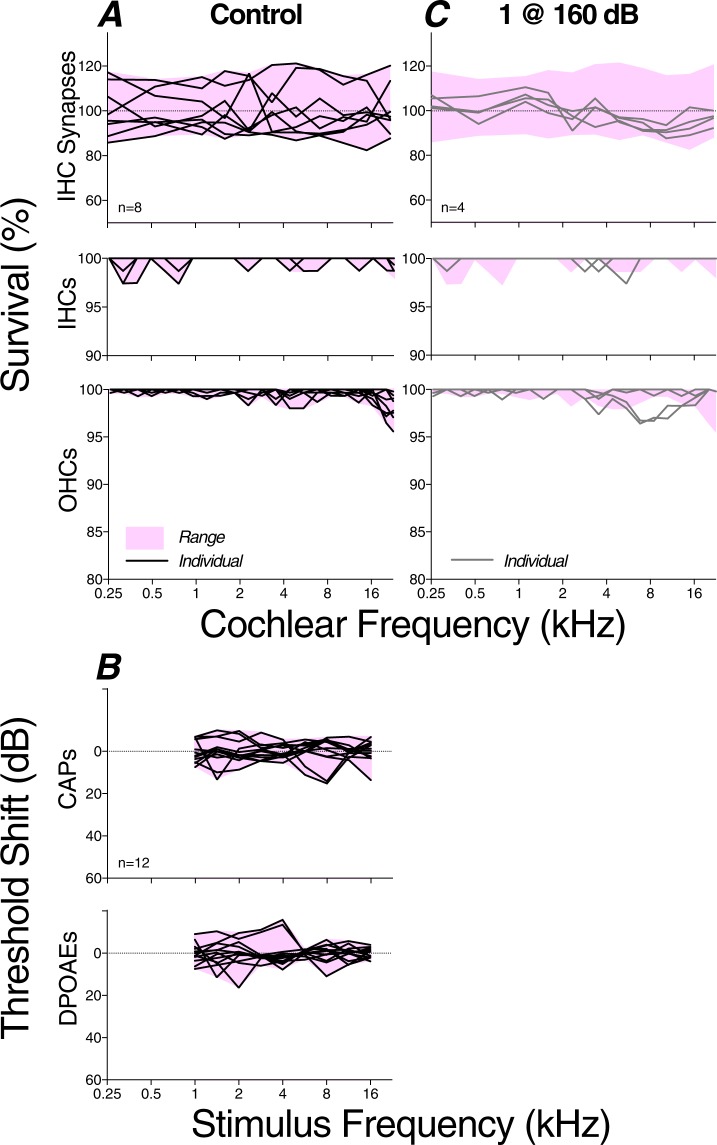
Summary histopathology (**A**,**C**) and physiology (**B**) from control ears (n = 8) and cases exposed to a single blast at the lowest SPL (n = 4; 160 dB peak). Data were obtained 1 wk post exposure. Each line in each panel is from a different case. Each IHC synapse value is derived from counts in two adjacent z-stacks, divided by the number of IHCs (including fractional values) in the same stacks. Each hair cell count is derived as shown in Fig. 3. Physiological measures were not obtained from this exposed group, thus there is no Panel D. All data are normalized to control means.

One week after exposure, animals were anesthetized for measurement of cochlear function via round window CAPs and ear canal DPOAEs (see Section C). Immediately after these physiological tests, cochleas were fixed and harvested for histological analysis. Complete cytocochleograms were assembled from hair cell counts in widefield images of organ of Corti wholemounts immunostained for myosin VIIa and binned into 50 equal-length segments along the 22 mm cochlear spiral (Fig. 3). As shown in Fig. 4A, in unexposed control ears, loss of outer hair cells (OHCs) or IHCs never exceeded 3% in any segment outside of the extreme base, where “gaps” in the regular hair cell lattice are more likely congenital than acquired.

**Figure 5:**
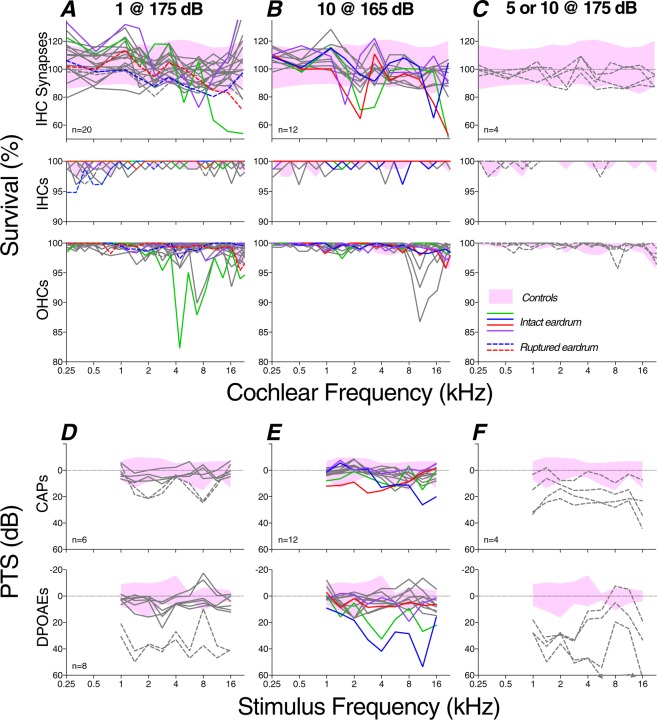
Summary histopathology (**A**–**C**) and pathophysiology (**D**–**F**) from blast groups exposed at the three higher SPLs and obtained 1 wk post exposure. Pink shading indicates the range of control values for each metric (see Fig. 4). Line colors are used to identify individual ears within each column, allowing direct comparison of pathophysiology and histopathology in the same ear. Cases with significant synaptopathy (i.e. fewer synapses than control mean by >2 standard deviations) are shown with colored lines; grey lines are for cases without significant synaptopathy. Dashed lines are for cases with ruptured eardrum. The key in C applies to all panels. All data are normalized to control means. All other aspects of data acquisition are as described in the caption for Fig. 4.

In exposed ears (Figs 4C and 5A–C), the segmental loss of IHCs never exceeded 6%, whereas focal loss of OHCs greater than 5% was seen in a subset of cases exposed to a single blast @ 175 dB (3/20) or to 10 blasts @ 165 dB peak (2/12). The absence of significant hair cell loss in cases exposed 5 or 10 times at 175 dB SPL (exposed vs. control p = 0.35 by repeated-measures 2-way ANOVA) is likely related to the eardrum ruptures observed in all these cases: the associated loss of middle-ear transmission is potentially protective to the inner ear and is reminiscent of observations in other multi-blast studies^18,24^.

**Figure 6:**
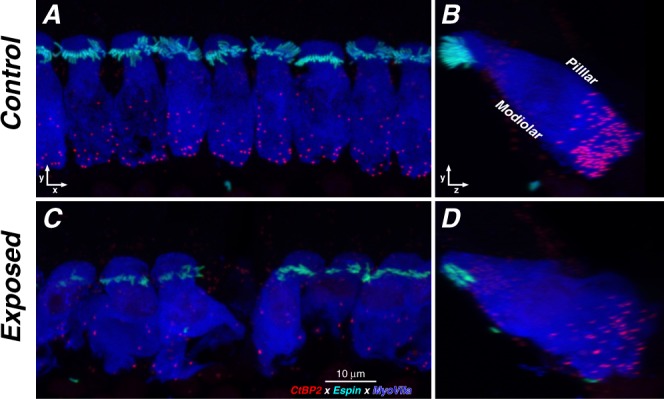
Some exposed ears showed striking loss of synaptic ribbons (CtBP2-red) in localized regions. (**A**,**C**) Confocal z-stacks from the 16 kHz region of a control and an exposed ear, 1 wk post blast, shown as maximum projections in the acquisition plane (xy). (**B**,**D**) The same z-stacks reprojected into the zy plane. The exposed ear also shows stereocilia damage (espin - cyan) and disruption of the normal modiolar-pillar gradient in ribbon size, which is normally correlated with the gradient of spontaneous rate and threshold in auditory-nerve fibers^28^.

To assess cochlear synaptopathy, the organ of Corti wholemounts were also immunostained for pre- and post-synaptic markers: anti-CtBP2 to label pre-synaptic ribbons within the hair cells, and anti-GluA2 to immunostain post-synaptic glutamate-receptor patches on the terminal dendrites of auditory-nerve fibers^32^. In a control ear (Fig. 6A), there are 15–20 pre-synaptic ribbons within the basal pole of each IHC, each representing a synaptic contact with a different auditory-nerve terminal^33^. When the z-stack is reprojected to view along an axis parallel to the cochlear spiral (Fig. 6B), the superimposed ribbon cloud from these 8+ IHCs shows the modiolar-pillar gradient of ribbon size (pillar side is closer to the OHCs), also reported in mice^28^ and guinea pigs^34^, that corresponds to the gradient in threshold and spontaneous rate in auditory-nerve responses^35,36^.

**Figure 7:**
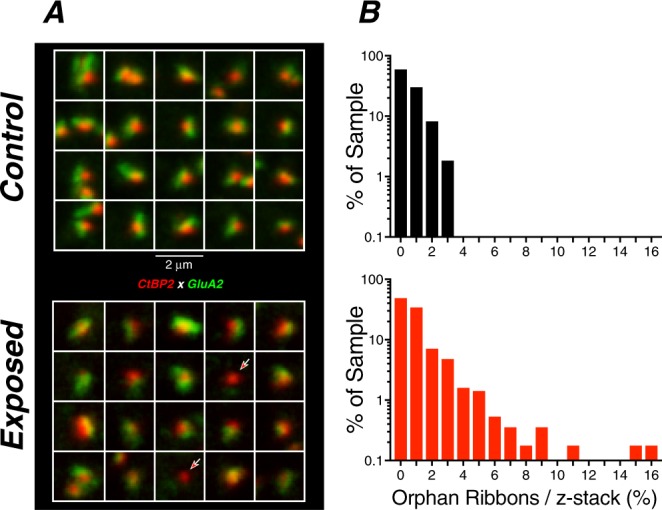
Orphan ribbons, lacking apposed glutamate receptor patches, were rare in blast-exposed ears. (**A**) Selected synaptic complexes from the control and exposed z-stacks shown in Fig. 6. Red arrows indicate orphan ribbons. (**B**) The histograms compare the % orphan ribbons per z-stack for all samples from 2–22 kHz, as compiled for all control (black) and exposed (red) cases. All data were gathered at 1 wk post blast.

In the place-matched image from the exposed ear (Fig. 6C), there is one missing IHC and some cytoplasmic distortion of the remaining IHCs, along with fewer synaptic ribbons per IHC, and a loss of the ribbon-size gradient normally observable in the zy projection (Fig. 6D). There also appears to be some damage to stereocilia bundles in the exposed ear. For clarity, these overview images do not show the confocal channel for the post-synaptic receptor patches. The apposition of pre- and post-synaptic elements in each case was assessed by assembling high-power thumbnail arrays of the voxel space around each ribbon (Fig. 7A). As observed in prior studies^37^, “orphan” ribbons, i.e. those unpaired with a glutamate receptor patch, were very rare (mean = 0.48%) in normal cases, and slightly, but significantly, more common (mean = 1.06%, p = 0.0045 by Welch’s t test) in exposed cases (Fig. 7B).

The degree of synaptopathy is summarized in each case in Figs 4A,C and 5A–C, where data are normalized to the mean control values at each cochlear frequency place, and can be compared to the hair-cell loss patterns in the same ears. The data suggest that, in cochlear regions near 2 kHz and 16 kHz, blast exposures can indeed cause significant cochlear synaptopathy (p = 0.0083 by Welch’s t test for controls vs. cases of 10 blasts @ 165 dB). Although the blasts sometimes caused focal OHC losses, the location and severity was uncorrelated with synaptopathy (r = −0.004, p = 0.9438 by Spearman’s correlation for cochlear regions from all cases of 10 blasts @ 165 dB or 1 blast @ 175 dB). The fact that synaptopathy and hair cell loss were virtually never seen in cases with tympanic-membrane rupture (dashed lines in Fig. 5) suggests that such disruptions of middle-ear sound transmission are protective for the delicate structures of the inner ear.

**Figure 8:**
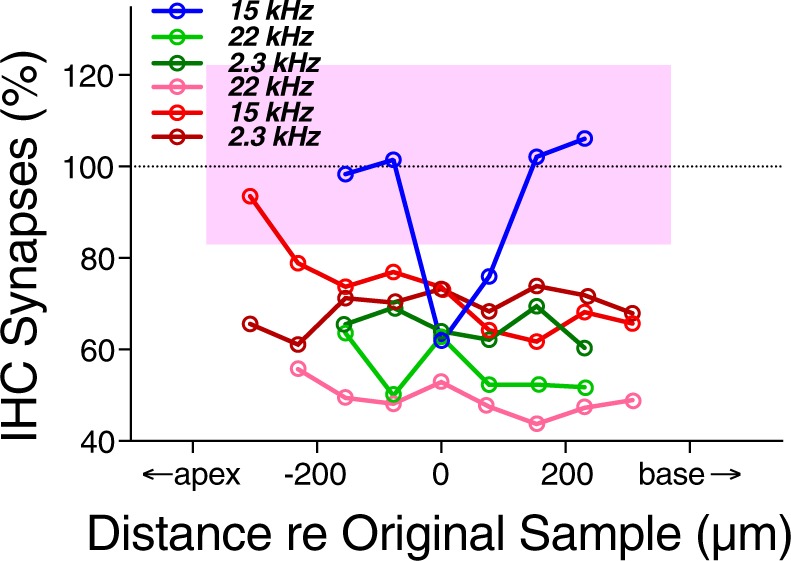
Most synaptopathic regions spanned at least 500 microns (~60 IHCs) along the cochlear spiral. For several of the synaptopathic points identified in the routine screen of half-octave positions along the cochlear spiral (e.g. Fig. 5B), we went back to the samples and imaged adjacent fields apical and basal to the initial sample. The case-identifying colors used here are the same as those in Fig. 5B; the key identifies the frequency region in question. The shaded pink region represents the combined control range from 2.3, 15, and 22 kHz.

Compared with the widespread regions of synaptopathy seen in prior studies after 2-hr exposures to octave-band noise^37^, the synaptopathy seen here in blast-exposed ears is more focal in nature. To further clarify how restricted these islands of synaptopathy are, we imaged serial adjacent fields in six of the most striking cases. As shown in Fig. 8, in 5/6 cases, the synaptic loss extended for several sample fields in either direction of the original imaging area; given an average IHC density of 10 cells/100 microns, these data show that the synaptopathic islands typically span at least 50–60 adjacent IHCs.

### Pathophysiology

**Figure 9:**
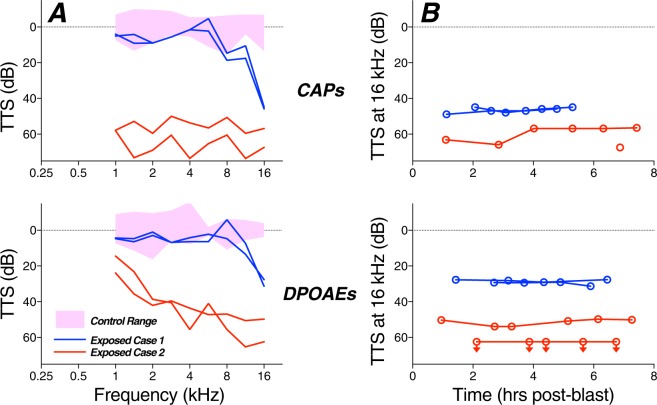
The amount of temporary threshold shift (TTS) was highly variable, as shown here for two ears from two animals exposed to 10 blasts @ 165 dB SPL. (**A**) Threshold shifts for CAPs and DPOAEs as measured 5.5–7.5 hrs after the blast. Control range (pink shading) is from Fig. 4B. (**B**) Threshold shifts at 16 kHz for each of the four animals at numerous post-blast time points show the stability of threshold shifts in this acute phase. Downward arrows indicate minimum estimates because thresholds were higher than those for system-generated distortion. One ear only had CAP measures at one post-blast timepoint. All data are normalized to control means.

One key aim of this study was to titrate the blast exposures to be “at the border” between reversible and irreversible threshold shift, to address the prevalence of synaptopathy in blast-exposed ears with “recovered” thresholds. As shown Fig. 5(D–F), the exposures we produced indeed straddled the border, in the sense that some ears showed no PTS, while others did. In two animals exposed to 10 blasts @ 165 dB SPL, we measured CAP and DPOAE thresholds for 0.75–8 hrs post exposure (Fig. 9): one animal showed threshold elevations restricted to the highest frequencies, where another showed large threshold shifts up to 50 dB throughout much of the frequency test range. Other studies of recovery from intense impulse noise in the chinchilla^23^ show that acute post-blast threshold shifts of as much as 60 dB can completely recover within 1 wk post exposure, as seems to have been the case for most of the cases here with intact eardrums.

Not surprisingly, ears with ruptured eardrums (dashed lines in Fig. 5) showed the most severe threshold shifts. The DPOAE threshold shifts were greater than those measured via CAPs, as expected because the DPOAEs are attenuated by changes in middle-ear forward and reverse transmission of stimulus and response, respectively, whereas the CAPs are only affected by forward transmission. The observation that DPOAE shifts were greater than CAP shifts in several ears without visible eardrum rupture (Fig. 5E) suggests there may have been other damage to middle-ear transmission, not visible from an otoscopic exam in the ear canal.

Prior studies have shown, after continuous-noise exposures at much lower SPLs (up to 116 dB), the DPOAE threshold shifts are always very close to the auditory brainstem response threshold shifts^38^, consistent with the idea that the functionally important structural changes in this type of noise-induced PTS are in the OHCs, likely in the form of stereocilia damage rather than hair cell death^21,22,39^. In the present study, the lack of correlation between OHC loss and CAP thresholds (r = 0.066, p = 0.457, Spearman’s correlation) or DPOAE thresholds (r = −0.119, p = 0.143, Spearman’s correlation) is not surprising, given the relatively small degree of OHC loss in the cases for which cochlear function was assessed.

It is also not surprising that we observed no correlation between synaptopathy and either CAP or DPOAE thresholds (r = 0.066, p = 0.454 for CAPs and r = −0.119, p = 0.143 for DPOAEs by Spearman’s correlation for all cochlear regions from all cases of 10 blasts @ 165 dB and 1 blast @ 175 dB), as prior studies observed that even greater degrees of neural loss do not affect cochlear thresholds^15,40^. We also evaluated CAP suprathreshold amplitudes at all the test frequencies (data not shown) and saw no significant differences between synaptopathic (10 blasts @ 165 dB) and control ears (repeated-measures 2-way ANOVA p = 0.864, Sidak multiple comparisons p > 0.62 at each test frequency).

## Discussion

### Middle ear damage: eardrum rupture and inner-ear protection

The middle ear appears to be among the most vulnerable structures in the human body to blast-wave exposure. In a study of 647 survivors of terrorist bombings in Israel^41^, 30% sustained primary blast injuries, and, among those, 90% showed eardrum rupture and 74% had no other injuries (hearing loss and tinnitus were not evaluated). Similarly, in a study of 94 survivors of the Boston Marathon Bombing^7^, 90% showed eardrum rupture. A retrospective review of >250 veterans of the “global war on terror” from 2003–2005 at Walter Reed^1^ found 32% with a history of eardrum rupture, and a similar review of 110 blast-injured veterans at the Naval Medical Center in San Diego from 2006–2007 reported eardrum rupture in 16% of patients^3^.

Blast waves of the type discussed above are extremely fast pressure transients, characterized by an almost instantaneous pressure rise followed by a very fast pressure fall and undershoot, and a return to atmospheric pressure, all within ~1–10 msecs^42^. This type of Friedlander wave is well mimicked by our compressed-air shock tube (Fig. 1). Peak pressures to which humans are exposed in these bombing contexts are not clear; however, experiments on cadaveric human temporal bones suggest that the “threshold” for eardrum rupture in humans is ~185 dB SPL peak (i.e. 35 kPa or ~5 psi), while ~50% of eardrums will rupture at 194 dB SPL^42^. Threshold for blast-wave lethality is said to be ~210 dB SPL (i.e. ~500 kPa or 72.5 psi).

Our experiments used blast pressures ≤178 dB SPL peak, because we wanted to assess cochlear function post-blast, and at ~175 dB SPL the eardrum sometimes ruptured even with a single blast (Figs 2 and 5A), and always ruptured after multiple blasts (Fig. 5C,F). For reference, although the blast durations may vary, a peak pressure of 165 dB would be that produced 50 ft from a hand-grenade explosion, and a peak pressure of 175 dB would be comparable to that experienced by a gunner firing an 8-inch howitzer^43^. Our data suggests that rupturing the eardrum is protective to the inner ear because none of the six ears with ruptured eardrums showed substantial loss of cochlear sensory cells (Fig. 5A,C). If the eardrum ruptures, less power will likely be delivered to the inner-ear fluids, since, in the absence of efficient middle-ear transmission, the pressure difference created across the cochlear partition will be reduced^18,44,45^. In another study including chinchillas exposed to repeated blasts, the ears with tympanic-membrane ruptures displayed less cochlear damage than ears with intact tympanic membranes^18^. Nevertheless, several human studies report no difference in audiometric thresholds in blast-exposed ears with vs. without eardrum rupture^46,47^. Interestingly, one study did find less sensorineural hearing loss on the ruptured vs. intact sides in cases of unilateral eardrum rupture^3^, despite the likelihood that the ruptured side experienced a higher peak SPL. It may be that intersubject differences in distance from the blast, physical orientation, and intrinsic “toughness” of the inner ear^48^ mask such protective effects of eardrum rupture in other human studies.

If eardrum rupture is protective, and if the human eardrum is tougher than the chinchilla eardrum, the human inner ear might be more vulnerable to blast-related cochlear injury. However, experiments with continuous-noise exposures suggest that the human inner ear may also be less vulnerable than that of small experimental mammals such as chinchillas^49^.

Even blasts that do not cause eardrum rupture visible on otoscopic examination can cause stiffness changes of up to 50% and structural damage visible in the scanning electron microscope^50^. Such changes help explain why several of the ears in the present study that showed no obvious eardrum rupture and no striking hair cell loss, nevertheless, showed large threshold shifts, and why those threshold shifts were sometimes greater in DPOAEs than in CAPs. Otoacoustic emissions are attenuated twice by middle ear damage, once as the stimulus is conducted from the ear canal to the inner ear and a second time when the response is reverse-transmitted from the cochlea to the ear canal.

### Inner-ear damage: cochlear synaptopathy and tinnitus

In a study of 94 survivors of the 2013 Boston Marathon Bombing, 68% reported new or worsening tinnitus, and patients with recovered audiograms nonetheless had persistent complaints of hyperacusis and difficulty hearing in noise^7^. A retrospective review of >250 veterans of the “global war on terror” from 2003–2005 at Walter Reed Hospital found 49% with tinnitus and average hearing thresholds that were significantly worse than expected for age-matched individuals without a history of acoustic overexposure^1^. A similar review of 110 blast-injured veterans at the Naval Medical Center in San Diego from 2006–2007 reported tinnitus in 55% of patients without eardrum rupture and 81% of patients with eardrum rupture^3^. Thus, in addition to increasing the risk for hearing loss, blast exposure may be one of the most reliable ways to elicit tinnitus in human subjects, and may also lead to hyperacusis and difficulty hearing in noise, even if thresholds return to normal.

This constellation of perceptual anomalies in blast-exposed ears with normal or near-normal threshold audiograms suggests that such ears might have “hidden hearing loss,” i.e. cochlear synaptopathy, a type of primary neural degeneration in the absence of hair cell loss. Numerous laboratories working in several animal models have shown that, for continuous-noise exposures, the synaptic connections between auditory-nerve fibers and hair cells are the most vulnerable elements in the inner ear^51^. Thus, continuous octave-band exposures at sound pressure levels near 100 dB SPL and durations of around 2 hrs can cause a transient threshold elevation that is nevertheless associated with a permanent loss of up to 50% of the synaptic connections between IHCs and auditory-nerve fibers^51^. This primary neural degeneration does not elevate threshold, because the loss is selective for that subset of auditory-nerve fibers with high thresholds and low spontaneous rates^34^, which are thought to be particularly important to stimulus coding in high levels of background noise^52^. It has been suggested that this peripheral deafferentation decreases the input to the auditory central nervous system and elicits a compensatory increase in “central gain,” which in turn leads to tinnitus and hyperacusis^16^. Indeed, spontaneous and sound-evoked neural hyperactivity has been observed in a variety of central auditory nuclei after acoustic overexposure^53^.

In the present study, we showed that similar vulnerabilities apply after blast-induced damage, i.e. that significant synaptopathy can be seen without significant threshold shift or hair cell damage. Since each auditory-nerve fiber contacts a single IHC via a single synaptic complex^33^, each missing synaptic puncta in the IHC area represents an auditory-nerve fiber that will no longer have spontaneous activity or response to sound. Despite a rich history of animal work on impulse-noise exposures designed to mimic workplace exposures to heavy machinery (e.g.^20^), there has been less laboratory work on the cochlear effects of blast waves, largely because of the practical difficulties in producing reproducible pressure transients that mimic explosive events in a laboratory setting. A recent mouse study^21^ showed that exposure to 199 dB SPL peak blasts routinely ruptured the eardrum and produced widespread loss of OHCs and >25 dB PTS, along with significant loss of synapses (on average ~40%) on surviving IHCs. This degree of synaptopathy in blast-exposed mice is similar to that seen in the present study in chinchillas (Fig. 8) and to that seen in mouse or chinchilla after continuous-noise exposures^12,51^, suggesting that it may again be the high-threshold fiber subset that is most vulnerable to blast. The similarity in degree of synaptopathy between exposures in mouse at 100 dB and 199 dB also suggests that there is a large vulnerability difference between the high-threshold and low-threshold subgroups of auditory-nerve fibers.

In the present study, synaptopathy was more focal in nature than that seen after continuous-noise exposures, where damage was detectable over cochlear span corresponding to 2 octaves of best frequency^10^. Here, we rarely saw synaptopathy in two adjacent 1/2 octave frequency samples (Figs 4 and 5). The creation of such localized regions of partial deafferentation may be key to the generation of a tonal (rather than noisy) tinnitus and may explain why tinnitus is such a reliable sequela of blast-induced damage.

There were two clear damage foci in our blast-exposed chinchillas (Figs 4 and 5). The mid-cochlear focus, centered near 2 kHz, may arise because of the acoustic filtering properties of the external ear canal, which shows a prominent mid-frequency peak that effectively raises the sound power reaching the inner ear in this mid-frequency region^54^. The basal focus at the extreme tip of the cochlear spiral is similar to that seen after continuous-noise exposure: high-frequency “tonopically inappropriate” lesions are often the first signs of permanent cochlear damage, regardless of the spectrum of the acoustic overexposure^55,56^. They are not explained by enhanced middle-ear transmission or cochlear mechanics at these high-frequency loci^57^, rather they likely arise because of inherent vulnerability gradients in the hair cells and neurons themselves.

Speculation about the mechanism of noise-induced synaptopathy has focused on glutamate excitotoxicity, as early work showed that the noise-induced swelling of auditory-nerve terminals seen immediately after acoustic overexposure could be minimized by cochlear perfusion of glutamate antagonists and mimicked by perfusion of glutamate agonists^58,59^. Although overwhelming the cochlear glutamate reuptake mechanisms is easy to imagine for synapses challenged with continuous-noise exposures lasting for hours, it is less obvious how the same mechanism could hold for a blast exposure lasting a few msec. However, the patterns of synaptopathy were indeed quite different for these two modes of acoustic overexposure, and it is possible that brief overstimulation of the IHC leads to an extended depolarization with associated high-levels of synaptic-vesicle release. Indeed, hair cell intracellular recordings in guinea pig have shown that even moderate levels of acoustic overstimulation can result in hair cell depolarizations that outlast the stimulus by many minutes^60^.

The time course and amplitude of the pressure waves from this study are similar to real-world explosive blasts and weapons fire. Thus, our findings of cochlear synaptopathy may help explain observed speech-in-noise deficits and tinnitus in “normal hearing” individuals exposed to impulsive noise in military, law-enforcement, hunting, or industrial settings^8,61^. Behavioral studies examining the duration and severity of tinnitus in animals exposed to synaptopathy-inducing blasts could help to further understand these common and debilitating pathologies. Since this study suggests eardrum rupture appears protective and that eardrum resilience may be a key determinant of blast-induced synaptopathy, further investigation of the role the middle ear plays in blast-induced cochlear trauma would help elucidate how similar impulses can cause such varied audiological outcomes in blast-exposed patients.
